# Field pea (*Pisum sativum* L.) shows genetic variation in phosphorus use efficiency in different P environments

**DOI:** 10.1038/s41598-020-75804-0

**Published:** 2020-11-03

**Authors:** Sarah Powers, Emily Mirsky, Anuruddha Bandaranayake, Pushparajah Thavarajah, Emerson Shipe, William Bridges, Dil Thavarajah

**Affiliations:** Plant and Environmental Sciences, 270 Poole Agricultural Center, Clemson University, Clemson, SC 29634 USA

**Keywords:** Genetics, Physiology, Plant sciences

## Abstract

Field pea is important to agriculture as a nutritionally dense legume, able to fix nitrogen from the atmosphere and supply it back to the soil. However, field pea requires more phosphorus (P) than other crops. Identifying field pea cultivars with high phosphorus use efficiency (PUE) is highly desirable for organic pulse crop biofortification. This study identified field pea accessions with high PUE by determining (1) the variation in P remobilization rate, (2) correlations between P and phytic acid (PA), and (3) broad-sense heritability estimates of P concentrations. Fifty field pea accessions were grown in a completely randomized design in a greenhouse with two replicates under normal (7551 ppm) and reduced (4459 ppm) P fertilizer conditions and harvested at two time points (mid-pod and full-pod). P concentrations ranged from 332 to 9520 ppm under normal P and from 83 to 8473 ppm under reduced P conditions across all tissues and both time points. Field pea accessions showed variation in remobilization rates, with PI 125840 and PI 137119 increasing remobilization of P under normal P conditions. Field pea accessions PI 411142 and PI 413683 increased P remobilization under the reduced P treatment. No correlation was evident between tissue P concentration and seed PA concentration (8–61 ppm). Finally, seed P concentration under limited P conditions was highly heritable (H^2^ = 0.85), as was mid-pod lower leaf P concentrations under normal P conditions (H^2^ = 0.81). In conclusion, breeding for PUE in field pea is possible by selecting for higher P remobilization accessions in low P soils with genetic and location sourcing.

## Introduction

The demand for organically produced crops is on the rise, with global retail sales reaching $81.6 billion in 2015; North America has the largest organic food market valued at $43.3 billion in 2017^1^. Consumers often cite the perceived transparency and sustainability of organic food production as their reason for buying organic crops, as organic agriculture has been shown to increase soil and plant health by using organic fertilizers and crop rotations with legumes^2,3^. However, organic fertilizers still utilize mined phosphorus (P) rock, which is a nonrenewable resource projected to run out within the century^4^. P is an essential nutrient required by all plants to grow, photosynthesize, and form proteins. It is especially limiting in organic environments for legumes, which need more P than cereals to form root nodules for nitrogen fixation^5,6^. Thus, identifying legumes that can acquire and efficiently utilize P from organic soils is highly desirable for organic agriculture^7–9^.

Field pea (*Pisum sativum* L.) is a pulse crop grown and consumed globally. Approximately 7.5 million hectares of field pea were harvested in 2018, with the top producers consisting of Canada, Russia, China, India, and Ukraine, followed by the United States^10^. Currently, field pea is increasing in popularity within the organic and health food markets, as it is a nutrient-dense crop, naturally rich in iron, zinc, prebiotic carbohydrates, and protein, ideal for animal feed and as an alternative protein source to animal products^11–13^. The superior nutritional value of field pea gives it the potential to combat ‘hidden hunger’, which is the global prevalence of micronutrient deficiencies due to cereal-dominated diets^14^. As such, increasing the production of field pea to diversify diets could help alleviate hidden hunger^15^ as well as benefit organic agriculture. However, as P is limiting to the growth of field pea and important for protein synthesis^6^, genotypes with strategies to better adapt to limited P soils should be investigated.

P is present as an inorganic form (P_i_) in low concentrations in soils (< 10 µm) and is highly immobile, often bound to Al and Fe ions making the P_i_ unavailable to the root^16–18^. Plants access and solubilize P_i_ by increasing root growth and organic acid exudation, thereby increasing transporter affinity for P_i_, as well as remodeling lipids as an internal P source^17,18^. Additionally, most vascular plants are able to form associations with arbuscular mycorrhizal fungi (AMF) through the release of strigolactones from the root under P deficient conditions, as this symbiosis directly increases root surface area and access to P_i_ in the soil^19^. Other microbes have also demonstrated the ability to alter P solubility, availability, and uptake in various plant species^20–24^. Once P_i_ is acquired from the soil by the root hair, most are transported into the nodule of field pea, which acts as a large P sink^25^. The remaining P is translocated and stored in vegetative tissues, such as mature leaves before senescence or P stress triggers remobilization to younger tissues and seeds. Once P_i_ is stored in the seed, it is often converted to phytic acid (PA), which acts as an antinutrient by binding to micronutrients such as Fe and Zn, thus decreasing their bioavailability. A low PA line of field pea has been created via chemical mutagenesis^26^, but such lines are not permitted in organic agriculture^3^. Efforts to increase phosphorus use efficiency in field pea must carefully consider how P is stored in the seed, as increasing PA concentration could negatively impact human health and biofortification efforts.

Phosphorus use efficiency (PUE) is the amount of P recovered from the soil that is then translocated, remobilized, and utilized for plant physiological processes. However, PUE is not well understood, as most research pertains to analyzing phosphorus acquisition efficiency (PAE), which focuses on identifying plants capable of greater P acquisition under P deficient conditions, commonly through alterations in root exudation and root system architecture. It is not enough to breed plants capable of acquiring greater P, because if the plant is unable to translocate P efficiently throughout the entire plant, it is of little use. Ideally, plants with greater PUE can effectively scavenge P from P-limited soils and then use it throughout the plant to sustain growth and yield, before storing P in the seed for germination^27^. Remobilization of P from mature tissues is the primary source of P for reproductive tissues, so field pea with an increased capacity to remobilize P from mature tissues should maintain yield and seed quality^12,27^. It remains to be investigated whether increasing P concentration in the seed causes an increase in PA, which could negate biofortification potential. Several studies have analyzed PUE in grain crops such as maize^28^, common bean^29^, wheat^30^, spring barley^31^, rice^32^, cowpea^33^, and *Brassica oleracea*^34^, but we are unaware of any conducted in field pea. We hypothesized that genetic variation for PUE traits and breeding potential exists for field pea. We tested our hypothesis by considering both normal (treatment 1) and reduced (treatment 2) P fertilizer conditions and (1) investigating variation in tissue P concentrations and remobilization rates, (2) quantifying the relationship between PA and P concentrations for biofortification purposes, and (3) measuring the heritability of P concentrations across tissues to determine the feasibility of breeding for PUE in field pea.

## Results

### Overall statistical differences

**Figure 1:**
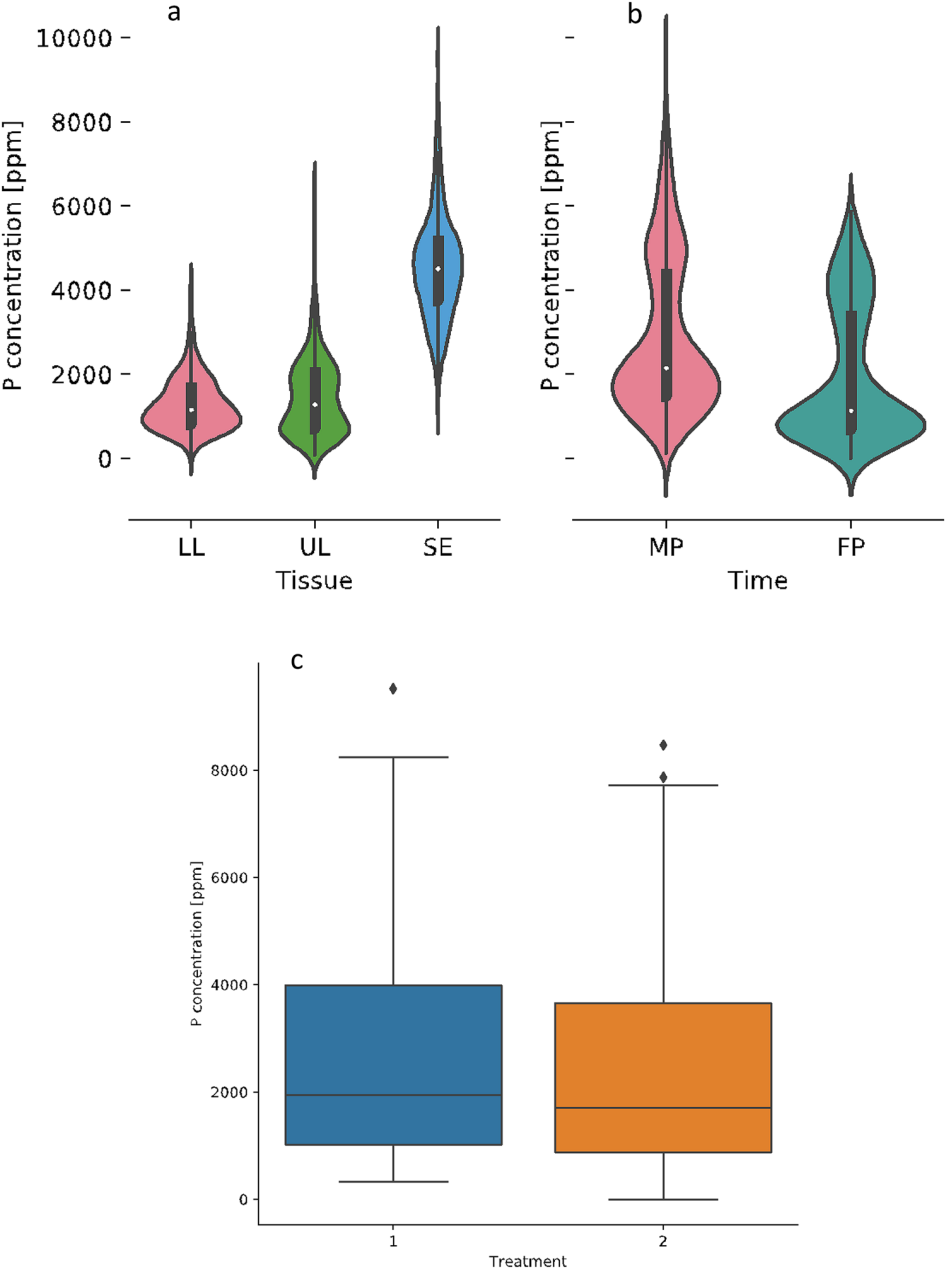
(**a**) Mean total P concentration in lower leaf (LL), upper leaf (UL), and seed (SE) tissues across both treatments. (**b**) Average P concentration at mid-pod (MP) and full-pod (FP). (**c**) Distribution of P concentrations across the normal P treatment (1) and low P treatment (2).

Analysis of variance indicates accession, treatment, time, and tissue play a significant (*p* < 0.05) role in determining the P concentration of pea plants (n = 52) (Table 1). Several interactions are also significant (*p* < 0.05) with respect to final P concentration: tissue × time, accession × tissue, accession × tissue × time, accession × time, and accession × treatment (Table 1). Means of all tissue P concentrations were not different between treatments (n = 406) (Fig. 1c). Seed P concentrations were much higher than other tissues at both mid-pod and full-pod time points (Fig. 1a,b).

**Table 1:** ANOVA for combined P concentration of leaf tissues and seeds.

Source of variation	DF	*p* value
Accession	51	< .0001**
Time	1	< .0001**
Tissue	2	< .0001**
Tissue × time	2	< .0001**
Accession × tissue	102	< .0001**
Treatment	1	< .0001**
Accession × tissue × time	102	0.0328**
Accession × time	51	0.0327**
Accession × treatment	51	0.18476
Error	102	0.8825

**Significant at *p* < 0.05.

### Genotypic effects

**Figure 2:**
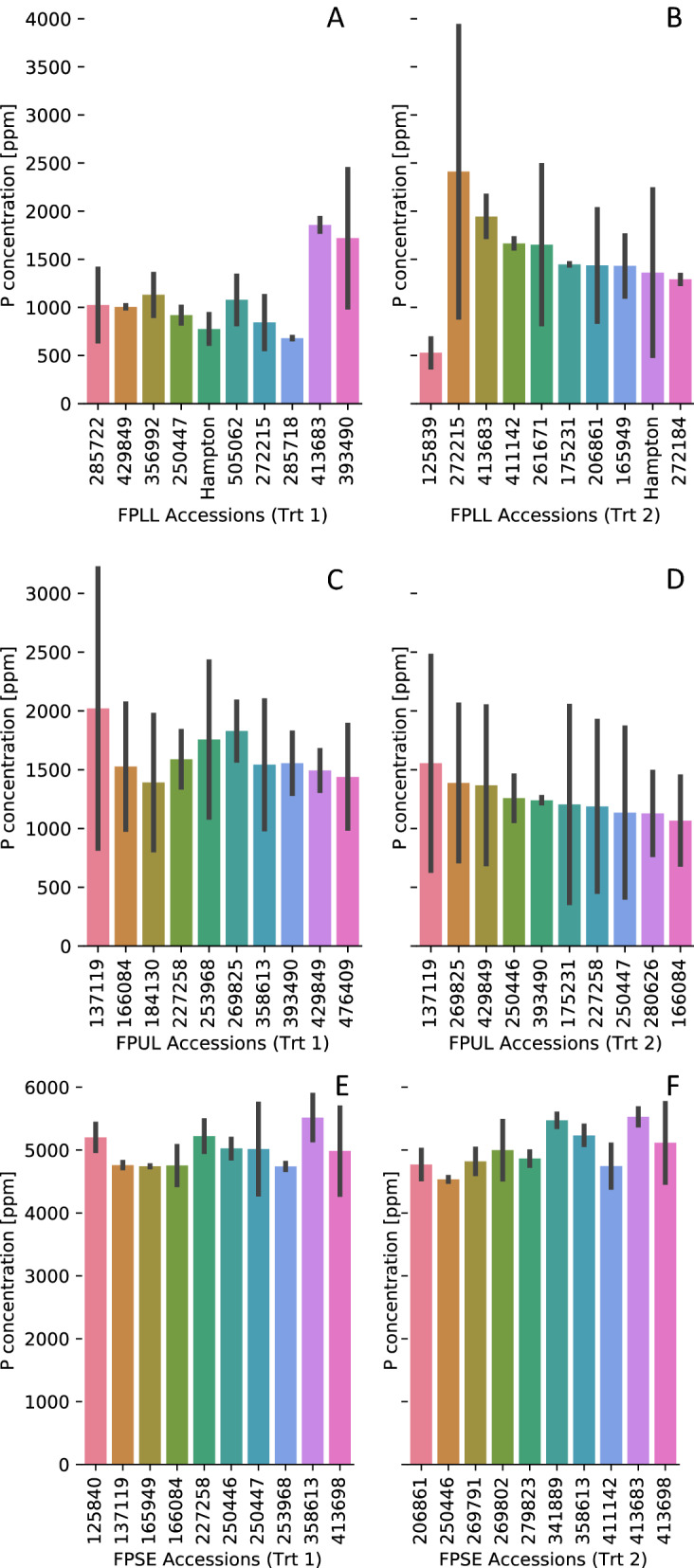
Variation in P concentration between tissues at full maturity for both treatments. The x-axes show the 10 accessions with the greatest P concentrations for treatment 1 (left: **A**, **C**, **E**) and treatment 2 (right: **B**, **D**, **F**) for full-pod lower leaf (FPLL) (**A**, **B**), full-pod upper leaf (FPUL) (**C**, **D**), and full-pod seed (FPSE) (**E**, **F**).

Field pea accessions significantly (*p* < 0.05) differ in terms of P tissue concentrations at both time points. Different accessions have the highest concentrations across tissues and treatments (Fig. 2). For treatment 1 (normal P), accessions 429849, 250447, 393490, 137119, 166084, 227258, 253968, and 358613 had the highest P concentrations in two of the three tissues. For treatment 2 (reduced P), accessions 413683, 411142, 175231, 206861, and 250446 had the highest P concentrations in two of the three tissues. Additionally, the accessions differed with respect to P resorption efficiency (PRE; range 40 to 100%) (Table 2), which is the ability to remobilize P to younger tissues (i.e., upper leaves and seeds) from that previously stored in mature tissues (lower leaves). Furthermore, P concentration in different tissues is heritable (Table 3), with full-pod seeds and mid-pod lower leaves showing the greatest broad-sense heritability estimates.

**Table 2:** Variation in accessions with respect to remobilization rate under normal and low P treatments.

Accession	Treatment^a^	PRE %	% Difference
125840	1	86.9	22.5
	2	64.5	
137119	1	87.7	19.3
	2	68.4	
179450	1	59.9	− 15.9
	2	75.8	
179970	1	92.2	52.3
	2	39.9	
250446	1	78.7	20.1
	2	58.7	
261671	1	56.6	− 34.9
	2	91.5	
280626	1	70.5	− 17.0
	2	87.5	
293426	1	93.1	33.1
	2	59.9	
393488	1	100.3	28.7
	2	71.6	
393490	1	87.2	29.6
	2	57.5	
411142	1	58.9	− 21.9
	2	80.9	
413683	1	55.5	− 36.0
	2	91.6	
Hampton	1	81.7	29.7
	2	51.9	
203069	1	96.4	− 1.9
	2	98.4	

^a^Treatments 1 and 2 correspond to normal and reduced P fertilizers, respectively.

**Table 3:** Broad-sense heritability of P concentration in tissues (lower leaf, LL; upper leaf, UL; seed, SE) at both time points (mid-pod, MP; full-pod, FP).

Tissue	Time	Treatment^a^	H^2^
LL	MP	1	0.81
LL	MP	2	0.60
SE	MP	1	0.57
SE	MP	2	0.26
UL	MP	1	0.63
UL	MP	2	0.15
LL	FP	1	0.56
LL	FP	2	0.40
SE	FP	1	0.66
SE	FP	2	0.85
UL	FP	1	0.13^b^
UL	FP	2	− 0.08

^a^Treatment 1 and treatment 2 correspond to normal and reduced phosphorus fertilizers, respectively.

^b^Variation influenced by replicate over accession.

### P treatment effects

Across both treatments, the mean P concentration of tissues (n = 208 × 2 replicates) is similar, at 2523 and 2326 ppm for treatment 1 (normal P) and treatment 2 (reduced P), respectively (Supplementary file 2). The P treatment is significant (*p* < 0.05) in determining P concentration (Table 1) but, overall, P concentrations appear to be similar between treatments (Fig. 1c). There was no significant genotype × environment (accession × treatment) interaction detected (Table 1). P treatment affects which accessions are able to accumulate the most P in various tissues (Fig. 2), as well as how much P different accessions are able to remobilize from their mature tissues (Table 2). PA and P concentrations in the full-pod seed were not correlated, and P treatment explains most of the variation observed in PA concentration (Supplementary file 4). Finally, most P concentrations were negatively influenced by the low P treatment and considered not heritable.

### Tissue and harvesting time effect

**Figure 3:**
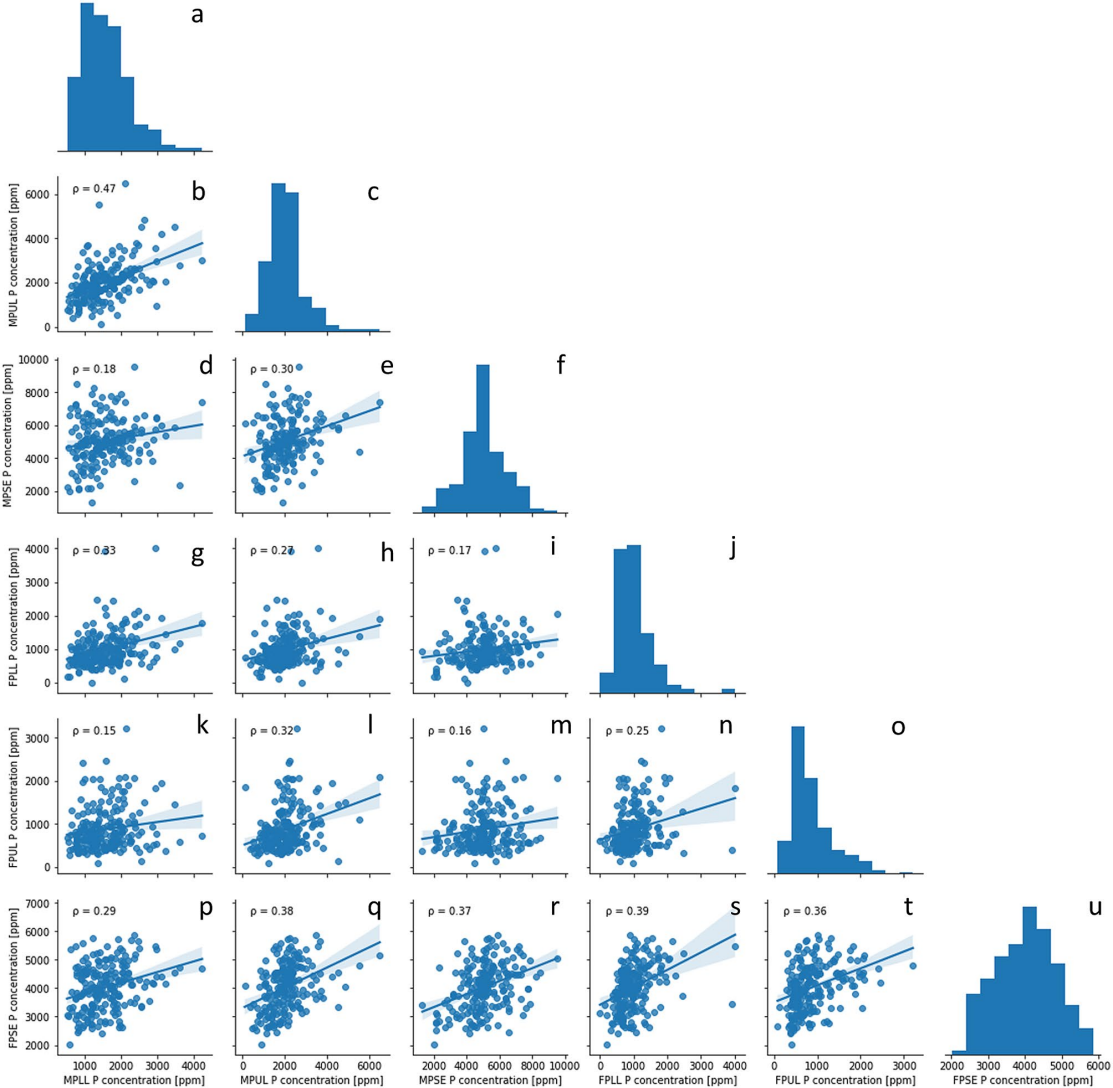
Correlations between P concentrations of different tissues at different time points. The histograms in **a**, **c**, **f**, **j**, **o**, and **u** indicate the distribution of mid-pod lower leaf (MPLL), mid-pod upper leaf (MPUL), mid-pod seed (MPSE), full-pod lower leaf (FPLL), full-pod upper leaf (FPUL), and full-pod seed (FPSE) P concentrations, respectively. The scatterplots show the joint distributions for P concentrations between two tissues to determine a correlation: MPLL vs. MPUL (**b**), MPLL vs. MPSE (**d**), MPUL vs. MPSE (**e**), MPLL vs. FPLL (**g**), MPUL vs. FPLL (**h**), MPSE vs. FPLL (**i**), MPLL vs. FPUL (**k**), MPUL vs. FPUL (**l**), MPSE vs. FPUL (**m**), FPLL vs. FPUL (**n**), MPLL vs. FPSE (**p**), MPUL vs. FPSE (**q**), MPSE vs FPSE (**r**), FPLL vs. FPSE (**s**), and FPUL vs. FPSE (**t**). The ρ values on the scatterplots indicate the correlation coefficient. The blue shaded regions on the scatterplots represent the 95% confidence intervals for each correlation.

Average P concentrations varied considerably between tissues, with the lower and upper leaves maintaining similar P levels and the seed containing greater P (Fig. 1a). Specifically, the mean P concentration of the lower and upper leaves (n = 416 × 2 replicates) was 1283 and 1457 ppm, respectively, and of the seed (n = 416 × 2 replicates) was 4533 ppm. However, a wide range of total P was found across accessions (Supplementary file 2). Total P in lower leaves ranged from 126 to 4233 ppm, which is similar to upper leaves that ranged from 83 to 6480 ppm. In contrast, seed P concentration ranged from 1312 to 9521 ppm. Total mean P concentration (n = 208 × 2 replicates) across time points was similar, at 2882 and 1967 ppm for mid- and full-pod, respectively. Overall, field peas at mid-pod had a higher concentration of P than plants at full-pod, for which a larger variation in concentration was noted. Correlations between P concentrations of tissues were generally weak (ρ < 0.4), with a moderate correlation (0.4 < ρ < 0.59) between mid-pod lower leaf (MPLL) vs. mid-pod upper leaf (MPUL) P concentration (Fig. 3b). The correlations between MPLL vs. MPUL (ρ = 0.47), MPUL vs. full-pod seed (FPSE) (ρ = 0.38) (Fig. 3q), mid-pod seed (MPSE) vs. FPSE (ρ = 0.37) (Fig. 3r), full-pod lower leaf (FPLL) vs. FPSE (ρ = 0.39) (Fig. 3s), and full-pod upper leaf (FPUL) vs. FPSE (ρ = 0.36) (Fig. 3t) were the highest of all tissues. Heritability estimates for P concentration differ between tissues and across the two time points, with FPSE and MPLL P concentration demonstrating consistent moderate to high heritability across treatments (Table 3).

## Discussion

Phosphorus is the most limiting nutrient in organic agriculture, especially for legumes such as field pea that require high amounts of P to sustain growth and form root nodules for nitrogen fixation^6^. Once P is acquired from the soil, it is stored in vegetative tissues (lower leaves) before the plant enters the reproductive stages, when the P is remobilized from lower leaves to younger tissues (upper leaves and seeds)^35^. Screening field pea germplasm for genetic variation in the ability to acquire and remobilize P to growing tissues under limited P environments is a promising strategy to identify accessions with greater P use efficiency. Additionally, a large portion of remobilized P will be stored in the seed as PA, an antinutrient that prevents the absorption of essential minerals from food in humans. Determining variation in field pea for lower PA concentrations is necessary to positively impact human health.

Field pea accessions varied in P concentration between lower leaves, upper leaves, and seeds (Fig. 1a) at the mid-pod and full-pod time points (Fig. 1b). Seeds consistently had the highest P concentration of all tissues, consistent with most P being stored in the seed as a reserve for germination^35^. Mean P concentrations of tissues were similar across treatments, but individual accessions differed in their response to P in the environment (Fig. 2). These results indicate plants must take up a basal amount of available P to sustain growth, and different acquisition strategies may be used by field pea under limited P conditions. For instance, genetic variation exists in several crop species (wheat^36^, white lupin^37^, rice^38^, maize^39^) for the ability to alter root morphology and organic acid exudation into the rhizosphere, which aids in solubilizing unavailable P in the soil. Additionally, field pea accessions may differ in the capacity to alter P transporter affinity in response to the environment^40^, contributing to the difference in accessions with the highest P concentrations between treatments (Fig. 2).

Field pea accessions also appear to differ for remobilization rates in normal or reduced P conditions (Table 2), which could contribute to higher seed P concentrations at reduced soil P (Fig. 2). For example, accession 137119 had one of the highest seed P concentrations in upper leaves and seeds under normal P conditions and was able to remobilize (PRE) approximately 88% of P from mature tissues. However, under reduced P conditions, the PRE was reduced to 68% and 137119 only had a high seed P concentration in the upper leaves. This same phenomenon was observed for accession 125840, which had a PRE of 87% for treatment 1 compared to 65% for treatment 2 and one of the highest seed P concentrations in treatment 1. Accessions 413683 and 411142 showed opposite trends to this, having higher seed P concentrations and PRE values of 91 and 80%, respectively, for treatment 2 compared to treatment 1. Interestingly, accession 250446 had high seed P concentrations for both treatments, even though the PRE was much less for treatment 2 (59 vs. 79%). This accession might be able to acquire more P than other accessions and translocate it to the seed. Finally, accession 203069 was included in Table 2 as it had the highest PRE for both treatments and seemed to acquire and remobilize P at equal rates across tissues, meaning it may be more P efficient compared to other accessions.

As seed P concentration requires a plant grown to full maturity and destructive analysis to phenotype, it is of great interest to develop a high-throughput method to determine seed P concentration that could be useful for a nutritional breeding program. From the correlation matrix in Fig. 3, seed P does not appear to be strongly correlated with P concentration in any other tissue. These results also indicate P concentrations among tissues are not dependent on each other, possibly relying more on environmental or genotype-dependent factors to supply P to the tissue. A moderate correlation (ρ = 0.47) between MPLL and MPUL indicates P concentration in the lower leaves somewhat corresponds to P concentrations in the upper leaves at mid-maturity. As mid-pod represents the early stages of P remobilization and seed filling, it is logical that P concentration throughout the plant would be equal. The correlations noted above for FPSE vs. MPUL, MPSE, FPLL, and FPUL (ρ = 0.36 to 0.39) are close to moderate, so it would be interesting to investigate these relationships further in a larger field pea population.

**Figure 4:**
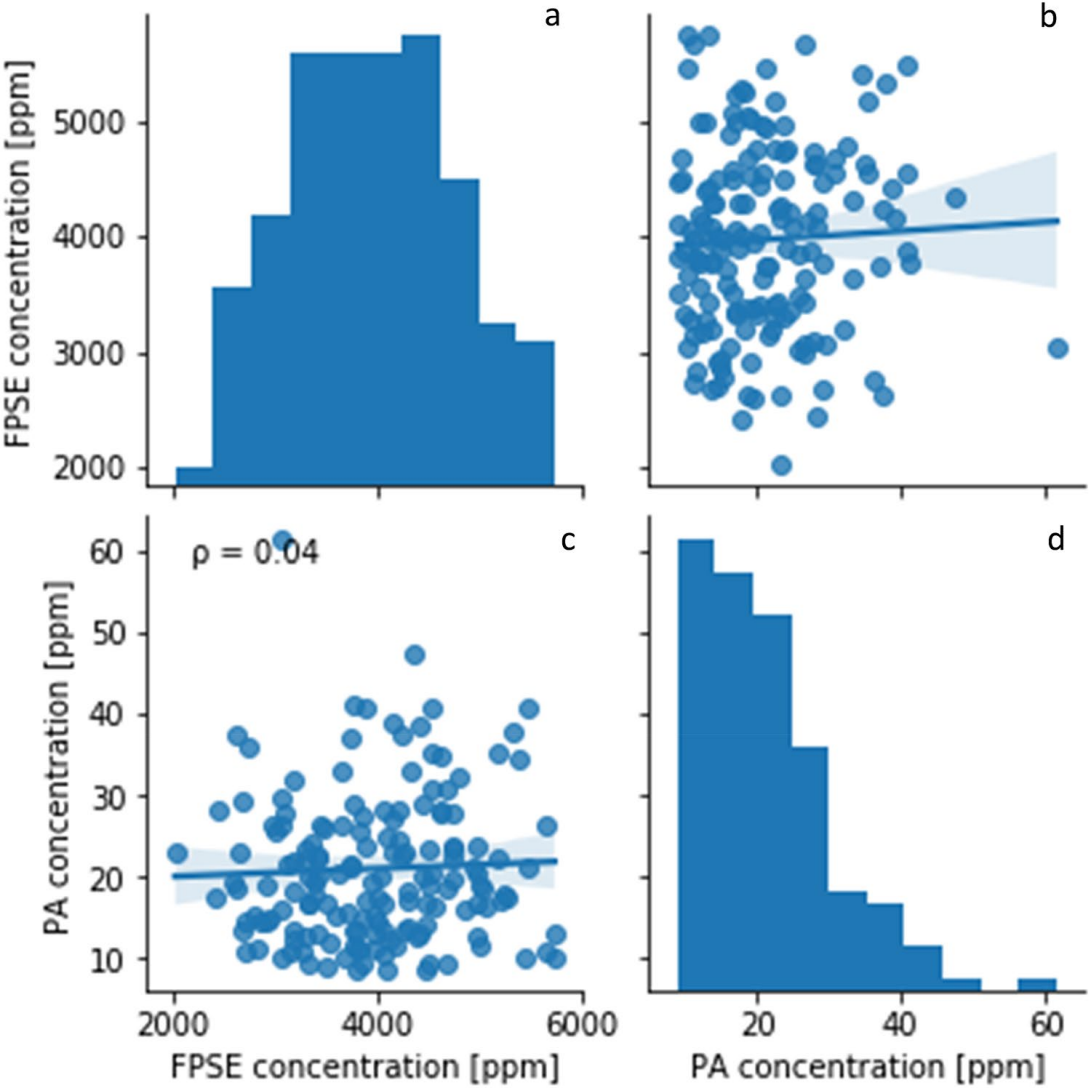
Average concentration of PA and full-pod seed (FPSE) P for all accessions and correlation of PA concentration to total P concentration. Plots (**a**) and (**d**) show the distribution of P and PA concentration across accessions, with the correlation between concentrations in (**c**). The inverse of the PA by P joint distribution is presented in (**b**). The ρ in c indicates the correlation coefficient. The blue shaded regions on the scatterplots represent the 95% confidence intervals for each correlation.

Evaluation of the breeding potential for lower PA seeds is a desirable target in the biofortification of field pea^41^. However, our analysis showed no genetic variation between accessions for PA concentration (Supplementary file 4) and that treatment may have the largest effect (Supplementary file 4). As PA conversion is a tightly conserved mechanism^42^, little variation should exist with respect to the genes controlling this process. Previous studies have demonstrated that alteration in the phytic acid pathway can generate plants with low phytic acid (lpa)^43^. While PA accumulation is under genetic control, there appear to be multiple mechanisms governing PA storage in seeds, as environmental and genotype x environment interactions are highly significant in determining PA concentration in rice, barley, and wheat^44–46^. No correlation was evident between the amount of P in the seed and PA concentration (Fig. 4), so considering PA content in studies of P use efficiency may not be necessary. Overall, these results indicate PA cannot be controlled through biofortification but only through environmental conditions. Several accessions had comparable or lower PA concentrations than those previously reported for low PA field pea mutants (Supplementary file 2)^26,47^. The PA concentration of several accessions was also lower than commercial lines (CDC Bronco and Hampton) (Supplementary file 2). Thus, low PA mutants may not be necessary to lower PA content in seeds, and natural physiological processes may exist that lower conversion rates. More analysis in a larger population will be needed to confirm these findings.

Finally, broad-sense heritability estimates for P concentrations across tissues and treatments determined that P concentrations in mid-pod lower leaves (H^2^ = 0.81 for treatment 1, H^2^ = 0.60 for treatment 2) and full-pod seeds (H^2^ = 0.66 for treatment 1, and H^2^ = 0.85 for treatment 2) were heritable under normal and limited P conditions (Table 3). To our knowledge, these are the first heritability estimates reported for P tissue concentrations in field pea. The P treatment affects heritability estimates due to the genotype × environment interaction of the accession and available P in the soil. These results are still promising, as P acquisition and remobilization traits may be heritable and useful for conventional breeding for a P use efficient field pea line. The finding that full-pod seed concentration has higher heritability under more limited P conditions is especially interesting, as breeding for low P tolerant lines is a goal related to P use efficiency. Selecting for a plant with an increased capacity to transport and store P in vegetative tissues, such as the lower leaves, and then efficient mobilization of that P during reproduction to the seed may be possible. A limitation of this study is that it was designed for observation of remobilization and storage in above ground tissues and does not take into account the effects of the nodule as a P sink. It will be necessary to incorporate nodulation and nitrogen fixation in response to P limited environments and the effect on P remobilization and storage in the future. Additionally, studies to examine genetic diversity for traits related to phosphorus acquisition efficiency, such as root exudates and modifications of root system architecture will aid in fully determining the mechanism of PUE. A complex interplay of genes is likely responsible for these processes, which will need to be further elucidated in genetic studies.

## Conclusions

Significant genetic variation is evident in field pea with respect to P concentrations between tissues at different stages of maturity under different P treatments. Field pea accessions better able to acquire and remobilize P to younger tissues and seeds to sustain growth under P-limited conditions could be used to develop more P use efficient field pea breeding lines. Additionally, several field pea accessions contain low amounts of PA in both treatments indicating they may be naturally low in PA, which positively impacts human health. Finally, P concentrations in mid-pod lower leaves and full-pod seeds appear to be heritable, so breeding field pea for P use efficiency in low P environments such as organic agriculture is possible.

## Materials and methods

### Plant material and growth conditions

Nutritional data for the Pea Single Plant Plus Collection (PSPPC)^48^ were obtained via GRIN (https://npgsweb.ars-grin.gov/gringlobal/method.aspx?id=492084). The 25 accessions with the highest and lowest seed P concentrations were selected for this experiment. In total, 50 accessions were obtained and replicated twice under two fertilizer treatments in the greenhouse, along with seed for commercial cultivars Hampton and CDC Bronco for comparison. Two plants per accession were grown in potting soil (SunGro Professional Growing Mix SKU: SUGR2375003; pH 6.4, 136 lbs/A P) under conditions of 16 h day and temperatures of 20–22/18 °C day/night. All pots were hand watered to 70–80% of pot capacity using distilled water. A week after planting, all plants were given 1/2 teaspoon of osomocote (14-14-14); an additional starter of 250 mL of Peter’s Professional 20-20-20 fertilizer was given 5 d later to each pot to provide adequate nutrition and ensure growth. Three weeks after planting, the two P fertilizer treatments were initiated. Treatment 1 employed Hoagland’s solution (0.2 M KH_2_PO_4_, 1 M KNO_3_, 1 M Ca(NO_3_)_2_·4H_2_O, 0.4 M MgSO_4_·7H_2_O, 0.57 g L^−1^ H_3_BO_3_, 0.36 g L^−1^ MnCl_2_·4H_2_O, 0.04 g L^−1^ ZnSO_4_·7H_2_O, 0.016 g L^−1^ CuSO_4_·5H_2_O, 0.003 g L^−1^ H_2_MoO_4_·H_2_O, and 0.1 M FeEDTA) to create normal phosphorus conditions, and treatment 2 employed a modified Hoagland’s solution (0.1 M KH_2_PO_4_) representing reduced phosphorus conditions. After 5 days, all plants exhibited nitrogen deficiency and were given Hoagland’s fertilizer without phosphorus to alleviate symptoms. Three weeks after the fertilizers were applied, an additional round of normal and reduced P treatments was administered. Nitrogen and phosphorus deficiencies were observed, so additional Peter’s Professional 20-20-20 fertilizer was provided so that seed formation was not affected. From then on, the low phosphorus fertilizer contained no KH_2_PO_4_ while the normal P fertilizer was formulated as noted above. Both fertilizer treatments were applied every week until one of the remaining plants reached full maturity and was ready for harvest, approximately 90 d after planting. 

### Harvest and sampling

Plants were harvested at both mid-pod and full pod. One plant per pot was harvested at mid-pod, corresponding to the mid-maturity stage, which is when pods contain 50% moisture, visible by the pod greenness and seeds that contain enough moisture to fill the pod. Mid-maturity is also when senescence begins in the lower leaves, so chlorophyll readings using a SPAD meter (SPAD-502Plus, Konica Minolta, Inc., Japan) were taken to confirm this stage was reached. Full pod or fully mature samples were taken once the pods turned brown, were dry, and the seeds hardened inside the pod. The shoot was cut at the soil and measurements made for fresh/dry weight (g), fresh/dry seed weight (g), pod number, height (in), lower and upper leaf chlorophyll, seed count, and growth stage. Samples of the lower and upper leaves were subjected to nutrient analysis. To determine upper vs. lower leaves, total nodes were counted and divided in half. Upper leaf and lower leaf samples were taken starting from the top or bottom of the plant, respectively, and collected until approximately 2 g of leaf were collected. The same tissue sampling technique was repeated as for the mid-pod stage as described above.

### P mineral analysis

Total P mineral concentrations at the mid- and full-pod stages were determined using a modified HNO_3_-H_2_O_2_ procedure^49^. Initially, 200 mg of leaf tissue were weighed out for an overnight digestion in 4 mL of concentrated nitric acid (70% HNO_3_), but the protocol was modified to 100 mg of tissue to better break down the leaf structure. The leaf samples were heated to 150 °C for 2 h, and then 4 mL of hydrochloric acid (70% HCl) were added to the solution and digestion continued for an additional hour. The solution was then filtered through Whatman paper (20–25 µm) and diluted to 10 mL with deionized H_2_O. Mineral concentrations were determined by inductively coupled plasma emission spectrometry (ICP-ES; ICP-6500 Duo, Thermo Fisher Scientific, Pittsburg, PA, USA). Standards made using 1000 mg L^−1^ stock solutions were serially diluted to produce calibration curves from 0.5 to 5.0 mg L^−1^. The solution detection limit was 80 µg L^−1^ for P. Measurements using this method were validated using lentil and peach as references. For seed nutrient analysis, seed samples were ground into a fine powder (UDY Cyclone Sample Mill, UDY Corporation, Fort Collins, CO; 4 mm filter) from which a 200 mg sample was used for digestion and ICP-ES analysis. Moisture content was analyzed from a subsample of 15 random accessions from each tissue and measured after drying at 50 °C for 3 h.

### Phosphorus resorption efficiency

PRE is the amount of P exported from the mature tissues before death^27^ and is indicative of P remobilization. All PRE values were calculated according to:

$$PRE = 100 - \left\lfloor {\frac{{\mu_{{{\text{P}}1}} - \mu_{{{\text{P}}2}} }}{{\mu_{{{\text{P}}1}} }}} \right\rfloor \times 100\%$$

where μ_P1_ and μ_P2_ are the P concentrations in all tissues for treatment 1 and 2, respectively.

### Phytic acid analysis

The full maturity seed samples were prepared using the modified PA extraction protocol from Talamond et al.^50^ and Thavarajah et al.^49^. A 100-mg sample of finely ground seed was weighed out into a 15-mL polystyrene conical tube (17 ± 120 mm) with a fitted cap. Ten mL of 0.5 M HCl were added to the tube and the solution heated with stirring for 5 min by immersing the tube into boiling (~ 100 °C) water. The solution was centrifuged at 4000×*g* for 3 min, and the supernatant transferred to another tube. The PA in the supernatant was decomplexed from other ions with the addition of 1.5 mL of 12 M HCl. A high-performance anion exchange chromatograph with a conductivity detector was used for PA analysis (ICS-5000 Dionex, Sunnyvale, CA, USA). The PA was separated using an Omnipac Pax-100 (8 µm) guard column (Dionex, Sunnyvale, CA, USA) and quantified by conductivity detection. The solvents used for gradient elution were 130 mM sodium hydroxide (A), deionized water-isopropanol (50:50, v/v) (B), and water (C). The flow rate of the gradient elution was 1.0 mL min^−1^ with a total run time of 10 min. Retention time and peak area were used to identify and quantify PA from the seed samples^49,50^. PA standards from 10 to 500 mg L^−1^ were used for calibration curves, with the detection limit set at 5 mg L^−1^. The error tolerance was < 0.1% for all laboratory samples. The PA phosphorus concentration was calculated using the weight ratio of P atoms per molecule of PA (1:3.56)^11^.

### Statistical analysis

The experimental design was a 2 × 2 × 4 factorial design with each replicate randomized for field pea accessions (n = 52). Replicates were considered random factors for analysis. Each analysis used mean replicate concentrations when indicated. For missing data points, JMP Pro 14 software (SAS Institute Inc., Cary, NC, USA) was used to predict a P concentration value for the tissue based off of concentrations in other replicates and tissues of the accession (Supplementary file 3). Significant differences for P concentration from factors including accession, treatment, time, and tissue were calculated using a one-way analysis of variance (ANOVA) with α = 0.05 and significance set at *p* < 0.05. The strength of linear relationships was assessed using the Pearson correlation coefficient in JMP Pro 14. Mixed model ANOVA and broad sense heritability estimates were also performed using JMP Pro 14.

## Supplementary information

Supplementary Legends.

Supplementary Information 1.

Supplementary Information 2.

Supplementary Information 3.
