# Supplementary material for: Field pea (Pisum sativum L.) shows genetic variation in phosphorus use efficiency in different P environments
Source: Sci Rep. 2020 Nov 3;10:18940. doi: 10.1038/s41598-020-75804-0 (PMC7641124; doi:10.1038/s41598-020-75804-0)
Supplement: Supplementary file 1 — Supplementary Legends. [file 41598_2020_75804_MOESM1_ESM.docx]

Legends for supplementary files

1. Phosphorus Raw data for PI accessions
2. Response P and statistical effect Summary
3. Response PA and statistical effect Summary
