# Supplementary material for: Field pea (Pisum sativum L.) shows genetic variation in phosphorus use efficiency in different P environments
Source: Sci Rep. 2020 Nov 3;10:18940. doi: 10.1038/s41598-020-75804-0 (PMC7641124; doi:10.1038/s41598-020-75804-0)
Supplement: Supplementary file 3 — Supplementary Information 2. [file 41598_2020_75804_MOESM3_ESM.docx]

**Response New P**

**Effect Summary**

| **Source** | **LogWorth** |  | **PValue** |
| --- | --- | --- | --- |
| TISSUE | 255.368 | 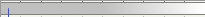 | 0.00000 |
| TIME | 41.750 | 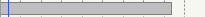 | 0.00000 |
| ACCESSION | 13.629 | 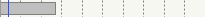 | 0.00000 |
| ACCESSION*TISSUE | 9.160 | 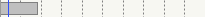 | 0.00000 |
| TISSUE*TIME | 8.480 | 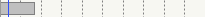 | 0.00000 |
| TREATMENT | 4.146 | 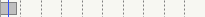 | 0.00007 |
| ACCESSION*TIME | 1.484 | 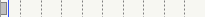 | 0.03279 |
| ACCESSION*TISSUE*TIME | 1.483 | 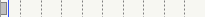 | 0.03286 |
| ACCESSION*TREATMENT | 0.733 | 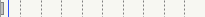 | 0.18476 |
| ACCESSION*TIME*TREATMENT | 0.673 | 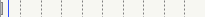 | 0.21219 |
| TISSUE*TIME*TREATMENT | 0.361 | 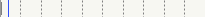 | 0.43524 |
| TIME*TREATMENT | 0.211 | 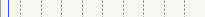 | 0.61552 |
| ACCESSION*TREATMENT*TISSUE | 0.171 | 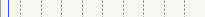 | 0.67387 |
| TREATMENT*TISSUE | 0.125 | 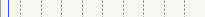 | 0.74980 |
| ACCESSION*TISSUE*TIME*TREATMENT | 0.054 | 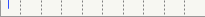 | 0.88253 |

**Summary of Fit**

| RSquare | 0.935396 |
| --- | --- |
| RSquare Adj | 0.870792 |
| Root Mean Square Error | 648.4863 |
| Mean of Response | 2417.803 |
| Observations (or Sum Wgts) | 1247 |

**REML Variance Component Estimates**

| **Random Effect** | **Var Ratio** | **Var Component** | **Std Error** | **95% Lower** | **95% Upper** | **Wald p-Value** | **Pct of Total** |
| --- | --- | --- | --- | --- | --- | --- | --- |
| REP | -0.002577 | -1083.754 | 155.52348 | -1388.574 | -778.9332 | <.0001* | 0.000 |
| ACCESSION*TREATMENT*REP | 0.0782115 | 32890.611 | 20001.761 | -6312.12 | 72093.343 | 0.1001 | 6.905 |
| ACCESSION*TREATMENT*REP*TIME | 0.0545233 | 22928.931 | 25008.852 | -26087.52 | 71945.379 | 0.3592 | 4.813 |
| Residual |  | 420534.44 | 29177.337 | 368732.72 | 484135.38 |  | 88.282 |
| Total |  | 476353.98 | 27587.957 | 426590.61 | 535410.56 |  | 100.000 |

-2 LogLikelihood = 11746.66505

Note: Total is the sum of the positive variance components.

Total including negative estimates = 475270.23

**Fixed Effect Tests**

| **Source** | **Nparm** | **DF** | **DFDen** | **F Ratio** | **Prob > F** |
| --- | --- | --- | --- | --- | --- |
| ACCESSION | 51 | 51 | 101.4 | 5.8450 | <.0001* |
| TIME | 1 | 1 | 100.6 | 547.0022 | <.0001* |
| TISSUE | 2 | 2 | 415.9 | 3307.748 | <.0001* |
| TISSUE*TIME | 2 | 2 | 415.9 | 20.4722 | <.0001* |
| ACCESSION*TISSUE | 102 | 102 | 415.7 | 2.3889 | <.0001* |
| TREATMENT | 1 | 1 | 101.4 | 17.1536 | <.0001* |
| ACCESSION*TISSUE*TIME | 102 | 102 | 415.7 | 1.3171 | 0.0329* |
| ACCESSION*TIME | 51 | 51 | 100.6 | 1.5432 | 0.0328* |
| ACCESSION*TREATMENT | 51 | 51 | 101.4 | 1.2334 | 0.1848 |
| ACCESSION*TISSUE*TIME*TREATMENT | 102 | 102 | 415.7 | 0.8232 | 0.8825 |
| TISSUE*TIME*TREATMENT | 2 | 2 | 415.9 | 0.8335 | 0.4352 |
| ACCESSION*TIME*TREATMENT | 51 | 51 | 100.6 | 1.2051 | 0.2122 |
| ACCESSION*TREATMENT*TISSUE | 102 | 102 | 415.7 | 0.9268 | 0.6739 |
| TIME*TREATMENT | 1 | 1 | 100.6 | 0.2538 | 0.6155 |
| TREATMENT*TISSUE | 2 | 2 | 415.9 | 0.2882 | 0.7498 |
