# Supplementary material for: Field pea (Pisum sativum L.) shows genetic variation in phosphorus use efficiency in different P environments
Source: Sci Rep. 2020 Nov 3;10:18940. doi: 10.1038/s41598-020-75804-0 (PMC7641124; doi:10.1038/s41598-020-75804-0)
Supplement: Supplementary file 4 — Supplementary Information 3. [file 41598_2020_75804_MOESM4_ESM.docx]

**Response PA**

**Effect Summary**

| **Source** | **LogWorth** |  | **PValue** |
| --- | --- | --- | --- |
| Treatment | 1.008 | 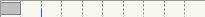 | 0.09809 |
| Rep | 0.462 | 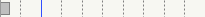 | 0.34499 |
| FPSE | 0.430 | 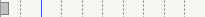 | 0.37114 |
| Accession | 0.189 | 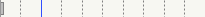 | 0.64667 |
| Accession*Rep | 0.180 | 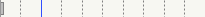 | 0.66102 |
| Accession*Treatment | 0.085 | 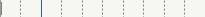 | 0.82253 |
| Rep*Treatment | 0.076 | 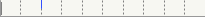 | 0.83989 |

**Summary of Fit**

| RSquare | 0.733484 |
| --- | --- |
| RSquare Adj | -0.16231 |
| Root Mean Square Error | 9.799051 |
| Mean of Response | 21.0519 |
| Observations (or Sum Wgts) | 158 |

**Analysis of Variance**

| **Source** | **DF** | **Sum of Squares** | **Mean Square** | **F Ratio** |
| --- | --- | --- | --- | --- |
| Model | 121 | 9513.454 | 78.6236 | 0.8188 |
| Error | 36 | 3456.770 | 96.0214 | **Prob > F** |
| C. Total | 157 | 12970.224 |  | 0.7891 |

**Effect Tests**

| **Source** | **Nparm** | **DF** | **Sum of Squares** | **F Ratio** | **Prob > F** |
| --- | --- | --- | --- | --- | --- |
| Accession | 39 | 39 | 3313.6347 | 0.8849 | 0.6467 |
| FPSE | 1 | 1 | 78.7572 | 0.8202 | 0.3711 |
| Treatment | 1 | 1 | 276.9343 | 2.8841 | 0.0981 |
| Rep | 1 | 1 | 87.9252 | 0.9157 | 0.3450 |
| Accession*Treatment | 39 | 39 | 2766.1886 | 0.7387 | 0.8225 |
| Accession*Rep | 39 | 39 | 3271.5199 | 0.8736 | 0.6610 |
| Rep*Treatment | 1 | 1 | 3.9764 | 0.0414 | 0.8399 |
